# Wasteosomes (*corpora amylacea*) of human brain can be phagocytosed and digested by macrophages

**DOI:** 10.1186/s13578-022-00915-2

**Published:** 2022-10-28

**Authors:** Marta Riba, Joan Campo-Sabariz, Iraida Tena, Laura Molina-Porcel, Teresa Ximelis, Maria Calvo, Ruth Ferrer, Raquel Martín-Venegas, Jaume del Valle, Jordi Vilaplana, Carme Pelegrí

**Affiliations:** 1 Secció de Fisiologia, Departament de Bioquímica i Fisiologia, Universitat de Barcelona, Av. Joan XXIII 27-31, 08028 Barcelona, Spain; 2 Institut de Neurociències, Universitat de Barcelona, Barcelona, Spain; 3 Centros de Biomedicina en Red de Enfermedades Neurodegenerativas (CIBERNED), Madrid, Spain; 4 Institut de Recerca en Nutrició i Seguretat Alimentàries (INSA-UB), Universitat de Barcelona, Barcelona, Spain; 5 Alzheimer’s Disease and Other Cognitive Disorders Unit, Institut d’Investigacions Biomèdiques August Pi i Sunyer (IDIBAPS), Neurology Service, Hospital Clinic, Universitat de Barcelona, Barcelona, Spain; 6 Neurological Tissue Bank of the Biobanc-Hospital Clinic-IDIBAPS, Barcelona, Spain; 7 Unitat de Microscòpia Òptica Avançada - Campus Clínic, Facultat de Medicina, Centres Científics i Tecnològics - Universitat de Barcelona, Barcelona, Spain

**Keywords:** Corpora amylacea, Wasteosome, Brain, Phagocytosis, Natural immunity, IgM, CD206, CD35, C3b

## Abstract

**Background:**

*Corpora amylacea* of human brain, recently renamed as wasteosomes, are granular structures that appear during aging and also accumulate in specific areas of the brain in neurodegenerative conditions. Acting as waste containers, wasteosomes are formed by polyglucosan aggregates that entrap and isolate toxic and waste substances of different origins. They are expelled from the brain to the cerebrospinal fluid (CSF), and can be phagocytosed by macrophages. In the present study, we analyze the phagocytosis of wasteosomes and the mechanisms involved in this process. Accordingly, we purified wasteosomes from post-mortem extracted human CSF and incubated them with THP-1 macrophages. Immunofluorescence staining and time-lapse recording techniques were performed to evaluate the phagocytosis. We also immunostained human hippocampal sections to study possible interactions between wasteosomes and macrophages at central nervous system interfaces.

**Results:**

We observed that the wasteosomes obtained from post-mortem extracted CSF are opsonized by MBL and the C3b complement protein. Moreover, we observed that CD206 and CD35 receptors may be involved in the phagocytosis of these wasteosomes by THP-1 macrophages. Once phagocytosed, wasteosomes become degraded and some of the resulting fractions can be exposed on the surface of macrophages and interchanged between different macrophages. However, brain tissue studies show that, in physiological conditions, CD206 but not CD35 receptors may be involved in the phagocytosis of wasteosomes.

**Conclusions:**

The present study indicates that macrophages have the machinery required to process and degrade wasteosomes, and that macrophages can interact in different ways with wasteosomes. In physiological conditions, the main mechanism involve CD206 receptors and M2 macrophages, which trigger the phagocytosis of wasteosomes without inducing inflammatory responses, thus avoiding tissue damage. However, altered wasteosomes like those obtained from post-mortem extracted CSF, which may exhibit waste elements, become opsonized by MBL and C3b, and so CD35 receptors constitute another possible mechanism of phagocytosis, leading in this case to inflammatory responses.

**Supplementary Information:**

The online version contains supplementary material available at 10.1186/s13578-022-00915-2.

## Background

C*orpora amylacea* of the human brain are polyglucosan aggregates that appear mainly in the periventricular, perivascular and subpial regions of the human brain during aging [1–3] and they also accumulate in specific areas of the brain in neurodegenerative conditions [4–7]. For a long time, they have been thought to entrap residual or waste products, including those derived from aging or degenerative processes [2, 3, 8–12], and they have recently been renamed as wasteosomes [13]. Although essentially composed of glucose polymers, wasteosomes can contain products derived from neurons, astrocytes, oligodendrocytes and the blood [4, 9, 14–19] or even related to viral, fungal or microbial infections [20–22]. Wasteosomes also contain ubiquitin and p62 proteins, which are both involved in the processing and collecting of waste products, as well as glycogen synthase (GS), which takes part in the formation of their polyglucosan structure [23]. Moreover, wasteosomes exhibit some neoantigens, more specifically neoepitopes (NEs), which are recognized by natural antibodies of the IgM isotype [10] and are of a carbohydrate nature [24]. NEs originate de novo and appear in both physiological and pathological conditions as well as in aging [25–29]. On the other hand, natural antibodies (including natural IgMs) are generated throughout a lifetime, even during fetal development and before external antigen exposure. They have been determined throughout evolution and are remarkably stable within species and even between species [30–35]. Natural antibodies are involved in the first line of immune defense against foreign microbes and also have important physiological functions [36]. Some of these natural antibodies are able to recognize some NEs that are present in, for example, cell remnants and senescent or apoptotic cells, participating in their controlled elimination and contributing to the maintenance of tissue homeostasis [26, 31, 33, 35–39]. The presence of NEs in wasteosomes and the existence of natural IgMs that target them reinforce the idea that wasteosomes are involved in brain cleaning or in protective processes [10]. Our group previously revealed that wasteosomes are released from the brain into the cerebrospinal fluid (CSF), some of them reaching the cervical lymph nodes via the meningeal lymphatic system. We also noticed that wasteosomes are phagocytosed in vitro by macrophages. All these findings indicated that wasteosomes not only entrap residual products, but they can also act as containers that participate in removing waste products from the brain [40].

Concerning the phagocytosis of wasteosomes, we previously observed that the wasteosomes obtained from the CSF and opsonized by IgMs are phagocytosed in vitro by macrophages derived from THP–1 cells (THP–1 macrophages). We also noticed that non-opsonized wasteosomes are phagocytosed as well, suggesting that the opsonization with IgMs is not required for the phagocytosis of wasteosomes [40]. Moreover, we detected the presence of the mannose receptor CD206 in THP-1 macrophages [40]. Liu et al. [41] suggested the presence of mannose in wasteosomes, as these structures are marked by concanavalin A (ConA), a plant lectin that recognizes certain carbohydrates, with a special affinity for mannose oligomers. Although the presence of mannose in wasteosomes needs to be verified, since ConA can bind to sugars other than mannose, this finding suggested that the phagocytosis of wasteosomes by THP–1 macrophages could involve the mannose receptor [40].

In this study, we aimed to shed light on the interactions between wasteosomes and THP–1 macrophages and to clarify the processes that trigger the phagocytosis of wasteosomes. Previous results have indicated that there may be redundant mechanisms that lead to the phagocytosis of wasteosomes [40]. Thus, we considered: (1) the possibility of the abovementioned mannose receptor pathway, in which THP–1 macrophages recognize the presence of mannose components in wasteosomes, (2) the process involving the opsonization of wasteosomes by IgMs combined with the presence of IgM receptors like FAIM3 (also known as FCμR) in THP–1 macrophages, and (3) the process involving the opsonization of wasteosomes with complement proteins (like the C3b protein) combined with the presence of complement protein receptors (like CD35) in THP–1 cells [42]. The activation of the complement system can occur via (a) the classical pathway, triggered by antibody-antigen interactions such as the interaction between IgMs and the NEs present in wasteosomes, (b) the lectin pathway, triggered by lectin-sugar interactions such as the interaction between mannose and mannose-binding lectin (MBL), and (c) the alternative pathway, in which complement proteins become activated directly upon contact with the particle to be engulfed.

In the present study, we also examined the responses generated by the phagocytosis of wasteosomes by macrophages. Once phagocytosed, wasteosomes might simply undergo a process of biochemical degradation, but it could also be possible that some of their components are exposed on the surfaces of macrophages, which would then act as antigen-presenting cells (APCs).

Besides in vitro studies, it is also of interest to elucidate the responses that are generated in vivo. The interaction between macrophages and wasteosomes cannot occur within the brain parenchyma in physiological circumstances as wasteosomes are intracytoplasmic astrocytic bodies and are therefore unreachable by infiltrating macrophages or even by microglia. However, this interaction could be possible in pathological conditions or inflammatory processes that induce tissue damage. In this sense, the phagocytosis of wasteosomes by macrophages has been described in the affected areas of tissue samples of the optic nerve and spinal cord in neuromyelitis optica [18]. Furthermore, given that wasteosomes are generally found in the perivascular, periventricular, or subpial regions of the brain and since they are released into the CSF and Virchow-Robin perivascular spaces [11, 40], it is conceivable that wasteosomes could be phagocytosed in these regions by meningeal macrophages, choroid plexus macrophages or perivascular macrophages [43]. This has not been previously examined and is also addressed in the present study.

For all these reasons, the objectives of the present work were to ascertain the mechanisms that trigger the phagocytosis of wasteosomes by THP–1 macrophages, find out how the wasteosomes are processed following phagocytosis by THP-1 macrophages and, finally, determine if macrophages located in the border areas or in the perivascular regions of the brain can interact with wasteosomes.

## Methods

### Studies on wasteosomes from human CSF samples

#### Human CSF samples

Post-mortem ventricular CSF samples were obtained from 7 neuropathologically affected patients (66 to 87 years old). When extracted, CSF samples were centrifuged at 4000×*g* at 4 °C for 10 min and the pellets obtained were stored at − 80 °C until use. All these procedures were performed at the Banc de Teixits Neurològics (Biobanc-Hospital Clínic-IDIBAPS, Barcelona). Medical data on these cases are detailed in Table 1.

**Table 1:** Medical data about the CSF donors

S	G	A	PMD	Clinical diagnosis^a^
1	M	66	08:00	Presenile AD
2	F	68	10:20	Presenile AD
3	F	69	07:08	ALS
4	M	71	12:00	AD VS LBD
5	M	74	06:40	MSA with Parkinsonism
6	F	87	07:10	PD

*S* subject, *G* gender (*F* female, *M* male). *A* age at death (years), *PMD* post-mortem delay (in hh:mm), ^a^*AD* Alzheimer’s Disease, *ALS* amyotrophic lateral sclerosis, *LBD* lewy body dementia, *MSA* multiple system atrophy, *PD* Parkinson’s Disease

All procedures involving human samples were performed in accordance with appropriate guidelines and regulations. All experiments involving human tissue were approved by the Bioethics Committee of the Universitat de Barcelona.

#### Wasteosomes obtaining and purification

Frozen pellets of CSF were defrosted and resuspended in 5 mL of phosphate-buffered saline (PBS) and divided into 500-µL aliquots in Eppendorf tubes. These aliquots were centrifuged at 700×*g* for 10 min, the supernatants were ruled out and the pellets obtained were resuspended in 1000 μL of PBS. This process was repeated 5 times to separate the wasteosomes from CSF cell debris and other remnants. Thereafter, another centrifugation at 700×*g* for 10 min was performed. The supernatants were ruled out and the wasteosomes were concentrated in the pellet for subsequent opsonization or staining.

#### ConA staining, AF555 NHS ester staining and IgM opsonization of wasteosomes

Purified wasteosomes for ConA staining or opsonization with IgMs were resuspended in 500 μL of Rhodamine-labeled ConA (ConA-Rhod; 1:250; RL-1002–25; Vector Laboratories), Fluorescein-labeled ConA (ConA-Fl; 1:250; FL-1001–25; Vector Laboratories) or purified human IgMs (1:10 dilution; OBT1524; AbD Serotec). Samples were maintained for 21 h at 4 °C with agitation. Purified wasteosomes for staining with Alexa Fluor (AF) 555 NHS ester (AF555-NHS; A37571; Thermo Fisher Scientific) were resuspended in 500 μL of 0.1 M sodium bicarbonate buffer (pH 8.3), while, in parallel, 100 µg of the amine-reactive dye AF555-NHS were dissolved in 10 µL of DMSO. Next, 10 µL of the AF555-NHS solution were slowly added to the sample. The mixture was then maintained for 1 h at room temperature under continuous stirring. After incubation with ConA, IgMs or AF555-NHS, samples were washed by centrifugation at 700 × *g* for 10 min, the resulting supernatants were ruled out and the pellets resuspended in 1000 μL of PBS. This washing process was repeated 3 times. Another centrifugation at 700×*g* for 10 min was performed and the supernatants were removed. The resulting pellets were then resuspended in supplemented RPMI 1640 media (Sigma-Aldrich) and added to the cell culture.

#### Periodic acid-Schiff (PAS) staining of wasteosomes

Purified wasteosomes were resuspended in 1000 µL of PBS and transferred to Pyrex glass tubes. Samples were centrifuged at 700×*g* for 10 min and the pellets obtained were resuspended in 1000 µL of periodic acid (0.25%; 9324–50; Electron Microscopy Sciences) and stirred for 5 min. Samples were centrifuged at 700 × *g* for 10 min, the resulting supernatants were removed and the pellets resuspended in 1000 μL of PBS. This washing process was repeated 3 times. Samples were centrifuged at 700×*g* for 10 min and the pellets obtained were resuspended in 1000 µL of the Schiff reagent (26052–06; Electron Microscopy Sciences) and stirred for 5 min. Another centrifugation at 700×*g* for 10 min was performed, the resulting supernatants were ruled out, and the pellets resuspended in 1000 μL of PBS. This three-step process was repeated 3 times. Finally, another centrifugation at 700×*g* for 10 min was performed and the supernatants were removed. The pellets were resuspended in supplemented RPMI and added to the cell culture.

#### Cell culture and differentiation

THP-1 cells provided by the American Type Culture Collection (ATCC) were subcultured at a density of 5 × 10^4^ cells/cm^2^ in 24-well plates with 12-mm round coverslips for the immunofluorescence studies and in 8-well chambered slides (µ-Slide 8 Well, Ibidi) for the time-lapse assays. Cells were differentiated into macrophages (THP-1 macrophages) with phorbol 12-myristate 13-acetate (PMA; Sigma-Aldrich) at a concentration of 100 nmol/L in RPMI supplemented with 10% heat-inactivated Fetal Bovine Serum (FBS) (GE Healthcare Life Sciences), 50 μM β-mercaptoethanol (Sigma-Aldrich), and penicillin (100 U/mL)/streptomycin (100 μg/mL) (Life Technologies) for 3 days. Differentiation of PMA-treated cells was enhanced after the initial 3-day stimulus by removing the PMA-containing media and incubating the cells in fresh supplemented RPMI for a further 3 days.

#### Phagocytosis studies: time-lapse assays

THP-1 macrophages were washed twice using supplemented RPMI without FBS. The cells were then stained for 30 min at 37 °C with 300 μL of the Vybrant^®^ CFDA-SE Cell Tracer Kit (1:2000; V12883; Thermo Fisher Scientific) in supplemented RPMI without FBS. Next, the cells were washed with PBS and incubated in 300 μL of RPMI with all the supplements. Before starting the time-lapse study, the stained macrophages were washed with supplemented RPMI 3 times before replacing the media with 300 μL of supplemented RPMI containing the wasteosomes stained with ConA-Rhod, AF555-NHS or PAS. The regions of interest (ROIs) were selected following specific criteria based on the presence and aspect of the wasteosomes and the closeness of the macrophages in relation to the chosen wasteosomes. By selecting the ROIs, only the X and Y coordinates were established. Three Z sections were appointed for each ROI. After selecting all the coordinates, the number of cycles was defined. A cycle consisted of the process of capturing every picture defined by the X, Y and Z coordinates. Thus, in each cycle, the number of pictures captured equaled the number of ROIs multiplied by the amount of Z coordinates selected for each field. In this study, the microscope took nearly 2 min to complete each cycle, which means that the frame rate of the definitive recording was 0.5 frames per minute (fpm). Once the sample was placed and the parameters established, the recording process was started and the microscope was left to record overnight for a minimum of 15 h. Automated multi-position live cell imaging was carried out using a Leica TCS SP5 (Leica Microsystems) or a Zeiss LSM 880 (Zeiss) confocal microscopes, both equipped with an Adaptive Focus Control system to keep the specimen in focus and an incubation system with controlled temperature (37 °C) and CO_2_ as well as a humidified atmosphere. Images of CFDA-SE (green channel) and ConA-Rhod, AF555-NHS or PAS staining (red channel) were acquired sequentially line by line using the 488 and 561 laser lines and detection ranges of 500–550 and 570–650 nm, respectively. All images were acquired using a Plan Apo 40 × oil immersion objective lens (NA 1.1) and a pinhole set at 1.5 Airy units. Simultaneously, bright-field images were acquired. The footage obtained was later processed using the software FIJI (National Institutes of Health, USA).

#### Phagocytosis studies: immunofluorescence assays

Purified and stained/opsonized wasteosomes obtained from the CSF and resuspended in 1000 μL of supplemented RPMI were added to the wells containing THP-1 macrophages. After 2 h, the macrophages were washed with Dulbecco’s PBS (DPBS; GIBCO) and fixed in 4% paraformaldehyde in PBS for 15 min. Samples were rehydrated with PBS and then blocked and permeabilized with 1% bovine serum albumin (BSA) in PBS (Sigma-Aldrich) (blocking buffer, BB) containing 0.1% Triton X-100 (Sigma-Aldrich) for 20 min. They were then washed with PBS and incubated for 21 h at 4 °C with one of the following primary antibodies: mouse monoclonal IgG_1_ against the mannose receptor CD206 (1:100; ab64693; Abcam), mouse monoclonal IgG_1_ against CD35 (1:40; MA5-13122; Thermo Fisher Scientific), mouse monoclonal IgG_1_ against CD68 (1:200; ab955; Abcam) or mouse monoclonal IgG_1_ against FcμR (1:150; MA5-13122; Thermo Fisher Scientific). Next, the samples were washed and incubated for 1 h at room temperature with the AF555 goat anti-mouse IgG_1_ secondary antibody (1:250; A-21127; Life Technologies) and the AF488 goat anti-human IgM heavy chain secondary antibody (1:200; A-21215; Life Technologies) if the wasteosomes were opsonized with human IgM. Nuclear staining was performed by incubating the samples with the Hoechst stain (2 μg/mL; H-33258; Fluka) for 5 min before washing and coverslipping them in ProLong Gold (Thermo Fisher Scientific). Staining controls were performed by incubating with the BB instead of the primary antibody.

#### MBL and C3b opsonization of wasteosomes and immunofluorescence assays

To opsonize wasteosomes with MBL and C3b, purified wasteosomes were resuspended in 500 μL of human plasma obtained from healthy donors from the Banc de Sang i Teixits de Barcelona and were maintained for 21 h at 37 °C under continuous stirring. Controls were resuspended in 500 μL of PBS instead of human plasma and maintained under the same conditions. Samples were washed by centrifugation at 700×*g* for 10 min, the resulting supernatants were ruled out and the pellets resuspended in 1000 μL of PBS. This washing process was repeated 3 times. Then, to evaluate the MBL or C3b attachment to the wasteosomes, samples were processed for immunofluorescence assays. Samples were centrifuged at 700×*g* for 10 min and the supernatants were removed. The resulting pellets containing the wasteosomes were incubated with 500 µL of the primary antibody, which was either the mouse monoclonal IgG_2b_ primary antibody against C3/C3b (anti-C3b; 1:100; ab11871; Abcam) or the FITC-conjugated rabbit polyclonal IgG antibody directed against MBL (anti-MBL; 1:100; orb463830; Biorbyt). Incubation with the primary antibody was maintained for 21 h at 4 °C with agitation. Staining controls were performed by incubating with 500 μL of PBS instead of the primary antibody under the same conditions. Once the incubations ended, the samples were washed by centrifugation at 700×*g* for 10 min, the resulting supernatants were ruled out and the pellets resuspended in 1000 μL of PBS. This washing process was repeated 3 times. Samples were centrifuged at 700×*g* for 10 min and the supernatants were removed. When using the unlabeled primary antibody against C3/C3b, the pellets were resuspended in 500 μL of the secondary antibody AF555 goat anti-mouse IgG_2b_ (1:250; A-21147; Life Technologies). The incubation was maintained for 1 h at room temperature with agitation. The samples were then washed by centrifugation at 700 × *g* for 10 min, the resulting supernatants removed and the pellets resuspended in 1000 μL of PBS. This process was repeated 3 times. The samples were then centrifuged at 700 g for 10 min and the supernatants were removed. Afterwards, the pellets obtained were resuspended in 40 μL of PBS, spread onto a microscope slide, air-dried and coverslipped with Fluoromount (Electron Microscopy Sciences) for microscope observation.

### Studies on wasteosomes from brain hippocampal sections

#### Human brain samples

Post-mortem brain samples were obtained from the Banc de Teixits Neurològics (Biobanc-Hospital Clínic-IDIBAPS, Barcelona). Frozen hippocampal Sects. (6 µm thick; stored at −80 °C) were obtained from two cases of neuropathologically confirmed Alzheimer’s disease and two cases of vascular encephalopathy. Medical data on these cases are detailed in Table 2.

**Table 2:** Medical data about the brain donors

S	G	A	PMD	Neuropathological diagnosis^a^
7	M	70	04:30	Aterosclerotic vascular encephalopathy
8	M	80	10:00	Vascular encephalopathy
9	M	86	07:15	AD (A2B1C1, Braak II, Thal 3, CERAD B) + LBD (Braak I, Brainstem)
10	M	89	12:00	AD (A3B3C3, Braak VI, Thal 5, CERAD frequent, severe CAA)

*S* subject, *G* gender (*F* female, *M* male), *A* age at death (years), *PMD* post-mortem delay (in hh:mm), ^a^*AD* Alzheimer’s Disease, *LBD* lewy body dementia

All procedures involving human samples were performed in accordance with appropriate guidelines and regulations. All experiments involving human tissue were approved by the Bioethics Committee of the Universitat de Barcelona.

#### Immunofluorescence studies of the hippocampal sections

Frozen hippocampal sections were left to defrost and air dry for 10 min at room temperature and then fixed with acetone at 4 °C for 10 min. After 2 h of further drying, the sections were rehydrated in PBS and then blocked and permeabilized with the BB containing 0.1% Triton X-100 (Sigma-Aldrich) for 20 min. They were then washed with PBS and incubated for 21 h at 4 °C with two primary antibodies for double staining. The primary antibodies used were: chicken polyclonal IgY against glial fibrillary acidic protein (GFAP) (1:300; AB5541; Merck), mouse monoclonal IgG_1_ against the mannose receptor CD206 (1:100; ab64693; Abcam), mouse monoclonal IgG_1_ against CD35 (1:40; MA5-13122; Thermo Fisher Scientific), mouse monoclonal IgG_1_ against CD68 (1:200; ab955; Abcam), mouse monoclonal IgG_1_ against FcμR (1:150; MA5-26353; Thermo Fisher Scientific), mouse monoclonal IgG_2a_ against p62 (1:400; ab56416; Abcam), mouse monoclonal IgG_2b_ against C3/C3b (1:100; ab11871; Abcam), FITC-conjugated rabbit polyclonal IgG antibody directed against MBL (1:100; orb463830; Biorbyt) and rabbit monoclonal IgG against GS (1:100; 15B1; Cell Signaling, Leiden, Netherlands). The slides were then washed and incubated for 1 h at room temperature with the corresponding secondary antibodies: AF488 goat anti-chicken IgY (H + L) (1:250; A-11039; Thermo Fisher Scientific), AF488 goat anti-mouse IgG_1_ (1:250; A-21121; Life Technologies), AF555 goat anti-mouse IgG_1_ (1:250; A-21127; Life Technologies), AF555 goat anti-mouse IgG_2a_ (1:250; A-21137; Life Technologies), AF555 goat anti-mouse IgG_2b_ (1:250; A-21147; Life Technologies) and AF488 donkey anti-rabbit IgG directed against both their heavy and their light chains (1:250; A-21206; Life Technologies). Nuclear staining was performed by incubation with the Hoechst stain (2 μg/mL; H-33258; Fluka) for 5 min and the slides were washed and coverslipped with Fluoromount (Electron Microscopy Sciences). Staining controls were performed by incubating with the BB instead of the primary antibody before incubation with the secondary antibody.

#### Image acquisition and processing

Hippocampal images were taken with a fluorescence laser and optical microscope (BX41, Olympus) and stored as tiff files. All the images were acquired using the same microscope, laser, and software settings. The exposure time was adapted to each staining, but the respective control images were acquired with the same exposure time. Image treatment and analysis were performed with ImageJ (NIH). Images that were modified for contrast and brightness to enhance their visualization were processed in the same way as those of their respective controls.

## Results

### Time-lapse study of the phagocytosis of wasteosomes by THP-1 macrophages

To study the phagocytosis of wasteosomes by THP-1 macrophages, different sets of experiments were performed using time-lapse imaging.

In the first set of experiments, wasteosomes obtained from the CSF were stained with ConA-Rhod and added to a culture of THP-1 macrophages that had been previously stained with the vital tracer CFDA-SE. From that point on and for each ROI (i.e., regions that contain wasteosomes), an image was taken every 2 min over a period of a minimum of 15 h. The sequence of images was then put into a video format to see the interaction between the macrophages and wasteosomes at each ROI during this period.

**Fig. 1:**
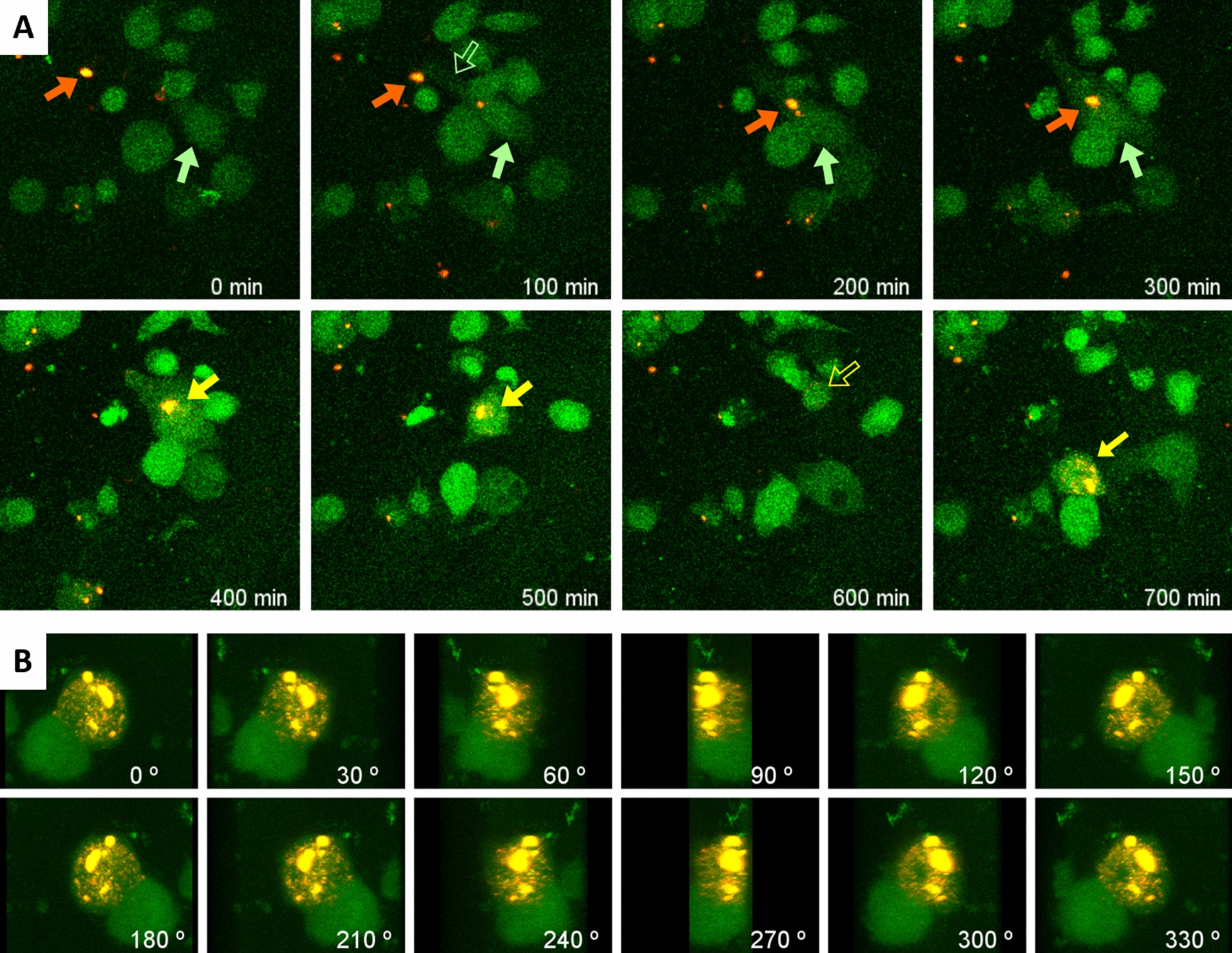
**A** Sequence of images from a time-lapse recording showing how a THP-1 macrophage (green arrow) extends a lamellipodium (empty green arrow) to a wasteosome opsonized with ConA (red arrow) and pulls it towards the body of the macrophage, triggering the engulfment of the wasteosome. The phagocytosed wasteosome become later digested and fragmented (yellow arrows). Empty yellow arrow: wasteosome is out of the focus plane. See video for details. At 1202 min, confocal images were taken and the 3D reconstruction was made. **B** Sequence of images showing the 360° rotation of the 3D reconstruction. Images permit to observe the location of the remains of the wasteosome (red and yellow) at the macrophage. Some dots of red or yellow fluorescence appear on the surface of the macrophage, suggesting antigen presentation. The big green spot in the lower region corresponds to a macrophage that is in contact with the one that has phagocytosed and digested the wasteosome

In all the ROIs, we observed that the macrophages did interact with wasteosomes. Additional file 1: Video 1A, summarized in Fig. 1A, shows the encounter of one macrophage with a wasteosome as well as several steps of phagocytosis. Firstly, a lamellipodium extends from the macrophage until it reaches the wasteosome. Once the lamellipodium has attached to the wasteosome, it pulls it towards the body of the macrophage and the macrophage completely engulfs the wasteosome. Once the wasteosome has been phagocytosed, the red fluorescence signal progressively spreads inside the macrophage, indicating that the wasteosome (or its ConA-Rhod protein fraction) has been digested. After the engulfment and digestion, some of the fluorescence appears on the surface of the macrophage. This process was corroborated by the 3D reconstructions from the confocal images obtained in the last moment of some sequences (Fig. 1B, Additional file 2: Video 1B). This suggests that parts of the stained wasteosome were exposed on the surfaces of THP-1 macrophages. Thus, THP-1 macrophages could act as APCs.

**Fig. 2:**
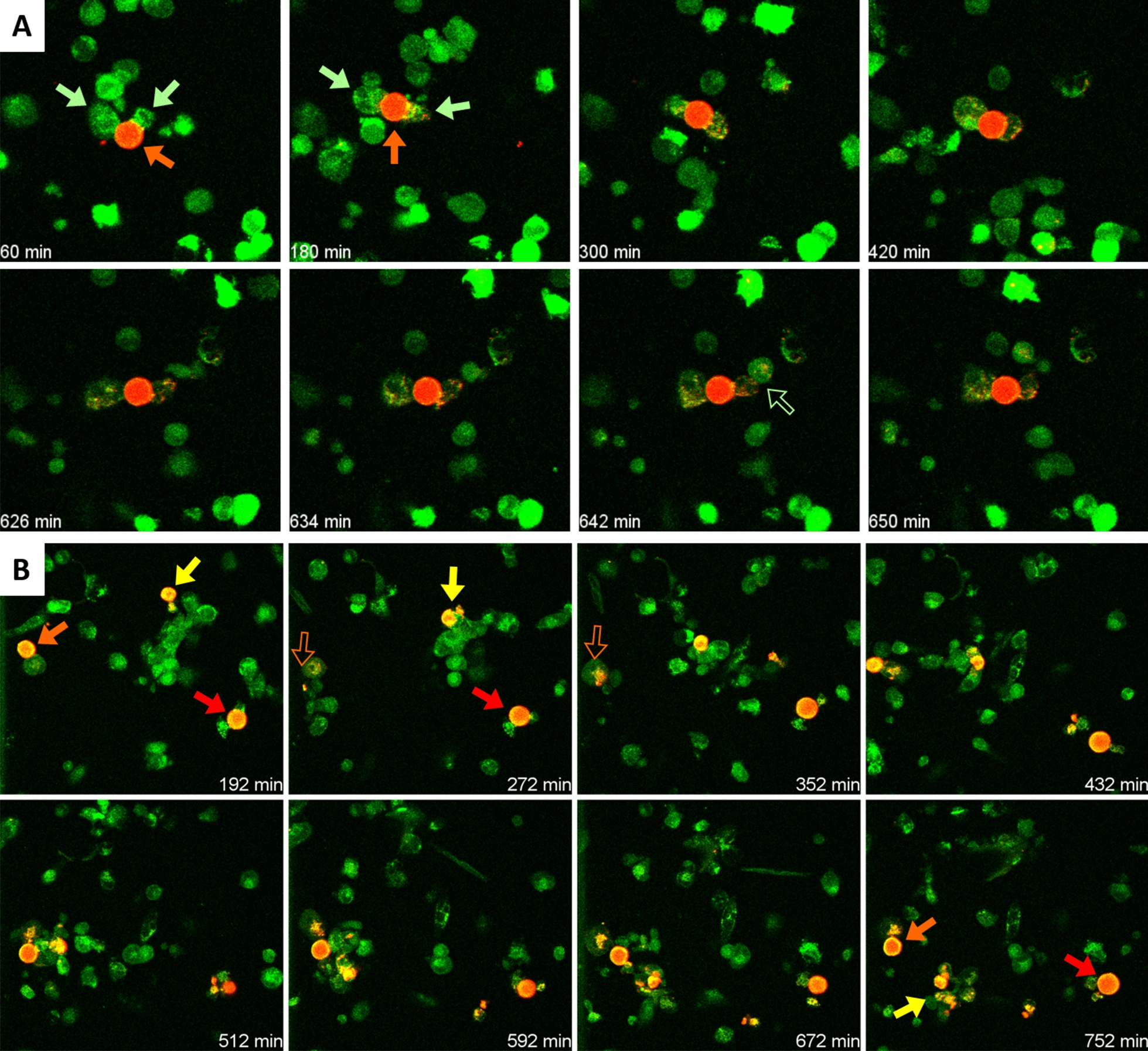
**A** Sequence of images from a time-lapse recording showing two THP-1 macrophages (green arrows) eroding a wasteosome opsonized with ConA (red arrow). Some spots of red fluorescence become incorporated into the macrophages. In some cases, the fluorescence is transferred from one macrophage to another one (empty green arrow). See video for details. **B** Sequence of images showing different macrophages interacting with three different wasteosomes (arrows). One of the wasteosomes (yellow arrow) become digested and fragmented. Empty arrows indicate that the wasteosome is out of the focus plane

In other cases, the wasteosomes were too big to be engulfed by the macrophages, but an interaction between the wasteosomes and macrophages could be observed. Figure 2A and Additional file 3: Video 2A show different macrophages making contact with a large wasteosome. The wasteosome is eroded by these macrophages and a gradual increase in the red fluorescence signal can be seen inside the macrophages. Moreover, some spots of fluorescence are also present on the surface of the macrophages and some interchanges of these spots of fluorescence between the different macrophages can be appreciated. Additional file 4: Video 2B and Fig. 2B show three different wasteosomes, of which two are just eroded by the macrophages and the remaining one is not just eroded but fragmented, with the resulting fragments digested by several macrophages.

From the results of this first set of experiments, we deduced that THP-1 macrophages can engulf and digest wasteosomes when these are opsonized by ConA-Rhod. Moreover, these macrophages might act as APCs by presenting the fluorescent component (i.e., ConA-Rhod or some of its fragments containing Rhod) on their surface.

In the second set of experiments, wasteosomes obtained from the CSF were stained with AF555-NHS and added to a culture of THP-1 macrophages that had been stained with CFDA-SE. In contrast to the first set of experiments, where we used an external protein (ConA-Rhod) that binds to the sugar components of wasteosomes, in this second set of experiments we used the AF555-NHS probe, which directly stains the proteins contained in the wasteosomes, thus enabling the determination of whether these proteins were also digested and presented on the surface of macrophages.

**Fig. 3:**
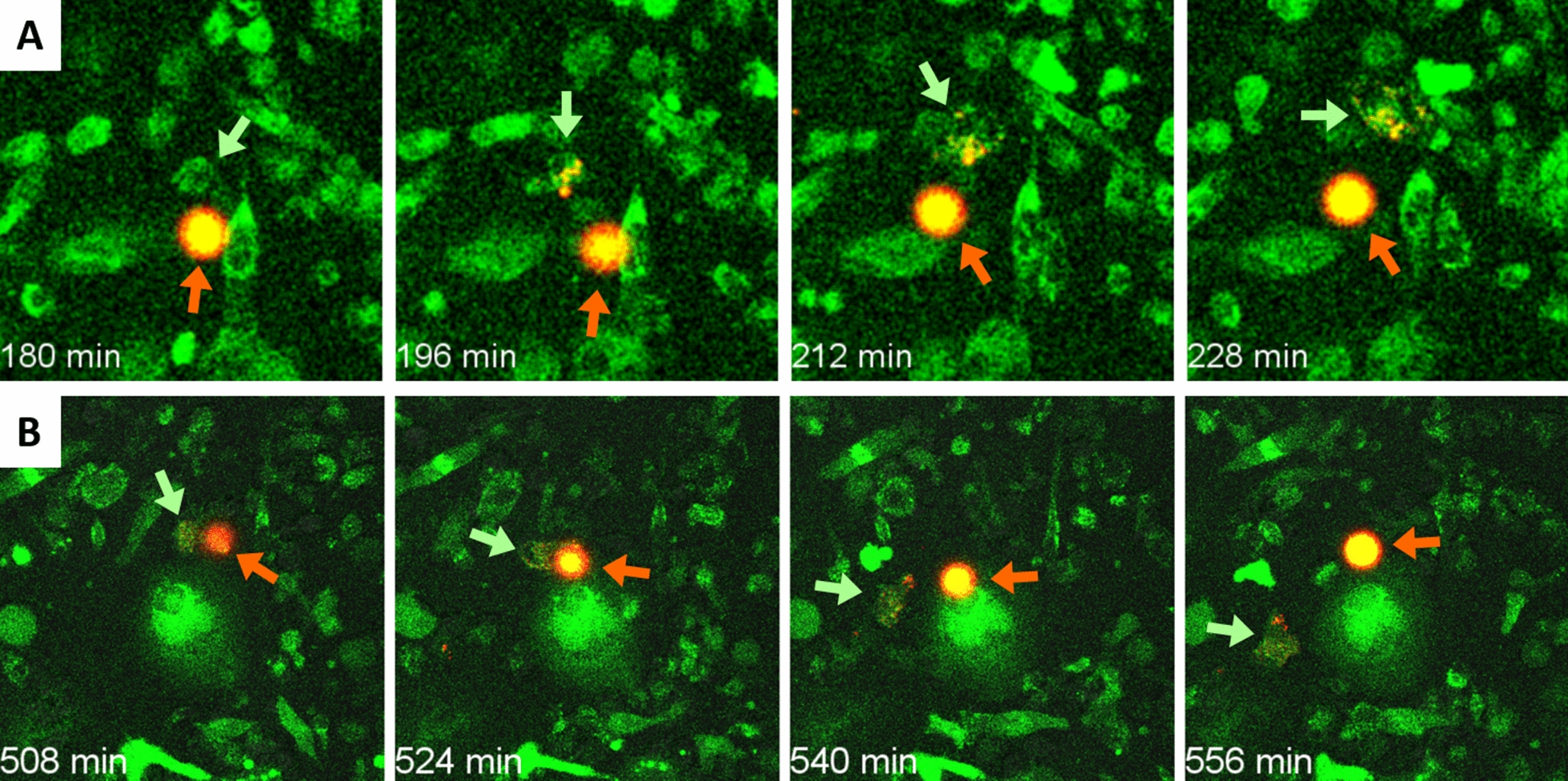
**A** Sequence of images showing a THP-1 macrophage (green arrow) eroding a wasteosome opsonized with AF555-NHS (red arrow). Note that the spots of red fluorescence, corresponding to stained proteins, become incorporated into the macrophage. A similar process can be observed in **B**. See the corresponding videos for details

In this second set of experiments, we also observed that some macrophages interacted with the wasteosomes stained with the AF555-NHS dye. Additional file 5: Video 3A and Fig. 3A show a macrophage making contact with a wasteosome at different times. Although the wasteosome is not entirely phagocytosed, some spots of red fluorescence are translocated from the wasteosome to the macrophage. When the macrophage detach from the wasteosome, the spots of fluorescence remain at the macrophage, indicating that the fluorescence signal from the wasteosome has been incorporated into the macrophage. The same process can be seen in Additional file 6: Video 3B and Fig. 3B. In this case, some spots of fluorescence can be observed on the surface of the macrophage, indicating possible antigen presentation. Thus, this second set of experiments indicated that the proteins contained in the wasteosomes can be phagocytosed by macrophages and that these proteins can later be presented on the surface of macrophages.

In the third set of experiments, wasteosomes obtained from the CSF were stained with the PAS technique and added to a culture of THP-1 macrophages that had been stained with CFDA-SE. In contrast to the first and second set of experiments, the fluorescence was generated here by the PAS staining, specifically related to the carbohydrate constituents of wasteosomes. This protocol allowed to determine if the carbohydrate components of wasteosomes are also digested and/or presented on the macrophage surface.

**Fig. 4:**
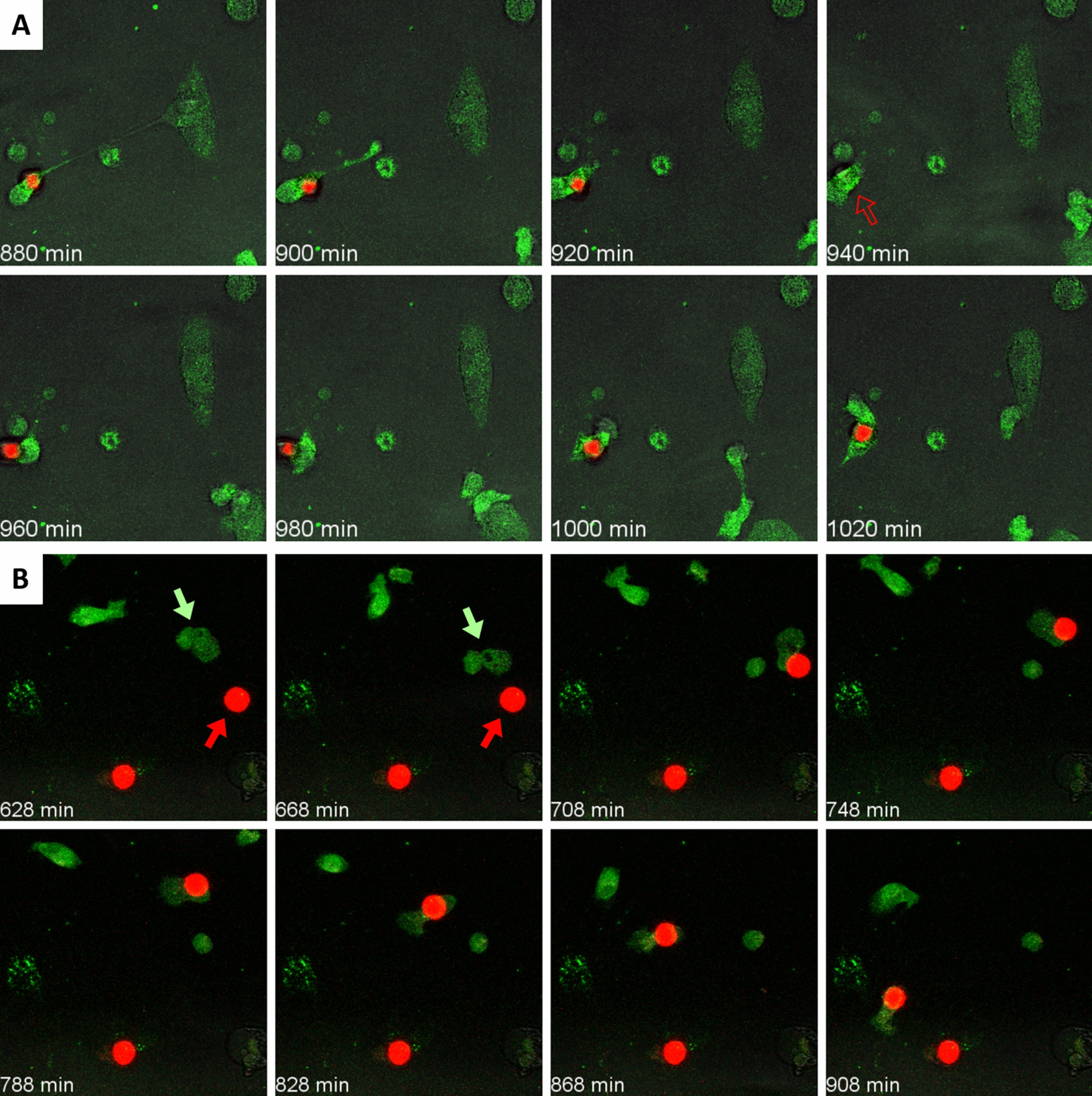
**A** Sequence of images showing a THP-1 macrophage (green) contacting a wasteosome stained with PAS (red). Initially, a lamellipodium from a distant macrophage making contact with the wasteosome can be observed. Thereafter, the macrophage shrinks over the wasteosome and both structures displace together. However, unlike in the previous sets of experiments, the digestion of the PAS stained wasteosomes could not be observed in most of the experiments. **B** In only a few cases, as the one shown here, small amounts of fluorescence were observed to detach from the wasteosomes and spread inside the macrophages. See videos for details

In this case, we observed that macrophages also interacted with wasteosomes. A representative sequence of images is presented in Additional file 7: Video 4A and Fig. 4A. Initially, a lamellipodium from a distant macrophage making contact with the wasteosome can be observed. Thereafter, the macrophage shrinks over the wasteosome and both structures displace together. However, unlike in the previous sets of experiments, digestion of the wasteosomes could not be observed in most of these experiments. In only a few cases, as that shown in Fig. 4B and Additional file 8: Video 4B, small amounts of fluorescence were observed to detach from the wasteosomes and spread inside the macrophages. In any case, the processes of phagocytosis, digestion and antigen presentation seemed to be reduced in this set of experiments, which indicates that the processing of the polyglucosan structure of wasteosomes differs from that of the protein fraction.

### Identification of phagocytic receptors on THP-1 macrophages that interact with wasteosomes

**Fig. 5:**
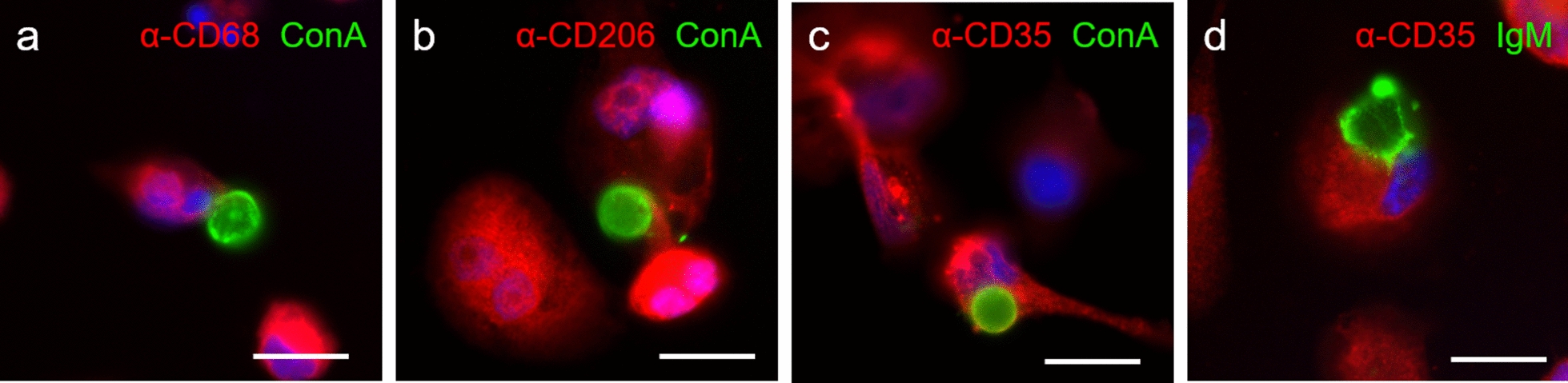
THP-1 macrophages which make contact with wasteosomes are CD68 + , CD206 + and CD35 + . **a** A CD68 + THP-1 macrophage (red) in contact with a wasteosome stained with ConA (green). **b** A CD206 + THP-1 macrophage (red) attached to a wasteosome (ConA, green). **c** A CD35 + THP-1 macrophage (red) encircling a wasteosome stained with ConA (green). **d** A CD35 + THP-1 macrophage (red) encircling a wasteosome immunostained with IgM (green). Nuclei are stained with Hoechst (blue). Scale bar: 25 µm

Since the time-lapse studies demonstrated that THP-1 macrophages interact with wasteosomes and can phagocytose them, the next step was to shed light on the mechanisms triggering this phagocytosis. In this regard, the study of the phenotype or, more specifically, of the phagocytic receptors expressed by THP-1 macrophages was determining. We postulated that the phagocytosis of wasteosomes could be mediated by CD206, CD35 and/or FAIM3. Thus, we isolated wasteosomes from the CSF and stained them with ConA-Fl or opsonized them with IgM before adding them to THP-1 macrophage cultures. The cultures were then fixed and processed for immunofluorescence analysis using anti-CD68, anti-CD206, anti-CD35 and anti-FcμR (directed against FAIM3) antibodies, and adding the AF488 anti-IgM antibody in the cases of wasteosomes that had been opsonized with IgM. This protocol allowed not only the detection of the abovementioned markers in THP-1 macrophages, but also the detection of these markers in wasteosomes-interacting macrophages. CD68 is a transmembrane glycoprotein that is highly expressed by human monocytes and macrophages [44]. It was used here to identify macrophages in the cultures, observe the interactions between macrophages and wasteosomes, and validate the effectiveness of the immunofluorescence method. As shown in Fig. 5a, THP-1 macrophages stained with the anti-CD68 antibody were observed to make contact with ConA-Fl-stained wasteosomes. Using the same protocol, we searched for the presence of the other indicated markers on macrophages that made contact with wasteosomes. CD206, also known as the mannose receptor, is a C-lectin that recognizes mannose residues, as well as N-acetylglucosamine and fucose residues [45]. It is normally expressed on M2, but not on M1 macrophages [46]. Figure 5b shows a representative image of CD206-positive THP-1 macrophage encircling and making contact with a ConA-Fl-stained wasteosome. These results, which are consistent with those obtained previously [40], suggest that macrophages that phagocytose wasteosomes are non-inflammatory or of the M2 subtype. Regarding the possible CD35-mediated phagocytosis, we stained THP-1 macrophages with the anti-CD35 antibody. CD35, also known as Complement Receptor type 1 (CR1), is a protein that binds to C3b/C4b-opsonized substances that are tagged for phagocytosis [47]. Figure 5c and d indicate that THP-1 macrophages that phagocytose wasteosomes express CD35, suggesting that these macrophages might also recognize some complement proteins in wasteosomes. Since wasteosomes are recognized by natural IgMs [10], complement activation could be triggered through the classical complement pathway, which would lead to wasteosomes opsonization by C3b and the induction of their CD35-mediated phagocytosis. However, as IgMs do not cross the blood–brain barrier, this process might occur in the lymphatic system or beyond, but not inside the brain. Since wasteosomes may contain mannose and N-acetylglucosamine, which are both targets of MBL [48], wasteosomes could also activate the complement system through the lectin pathway. The alternative pathway is the third biochemical pathway of the complement cascade and should also be considered. This pathway is based on the spontaneous hydrolysis of C3 into C3b. It has been reported that C3b binds to glucose oligomers [49, 50]. Since glucose has been described to be the main component of wasteosomes, C3b could probably bind to this carbohydrate. Given that natural IgMs recognize wasteosomes, we also considered FAIM3-mediated phagocytosis as well. FAIM3, also known as FcμR, is an IgM receptor found in some macrophages and dendritic cells that are associated with phagocytic processes [51–53]. Accordingly, we stained THP-1 macrophages with the anti-FcµR antibody, but these cells did not stain with this antibody, thus ruling out this pathway in the phagocytosis of wasteosomes by THP-1 macrophages.

### The presence of opsonins on the wasteosomes surface

**Fig. 6:**
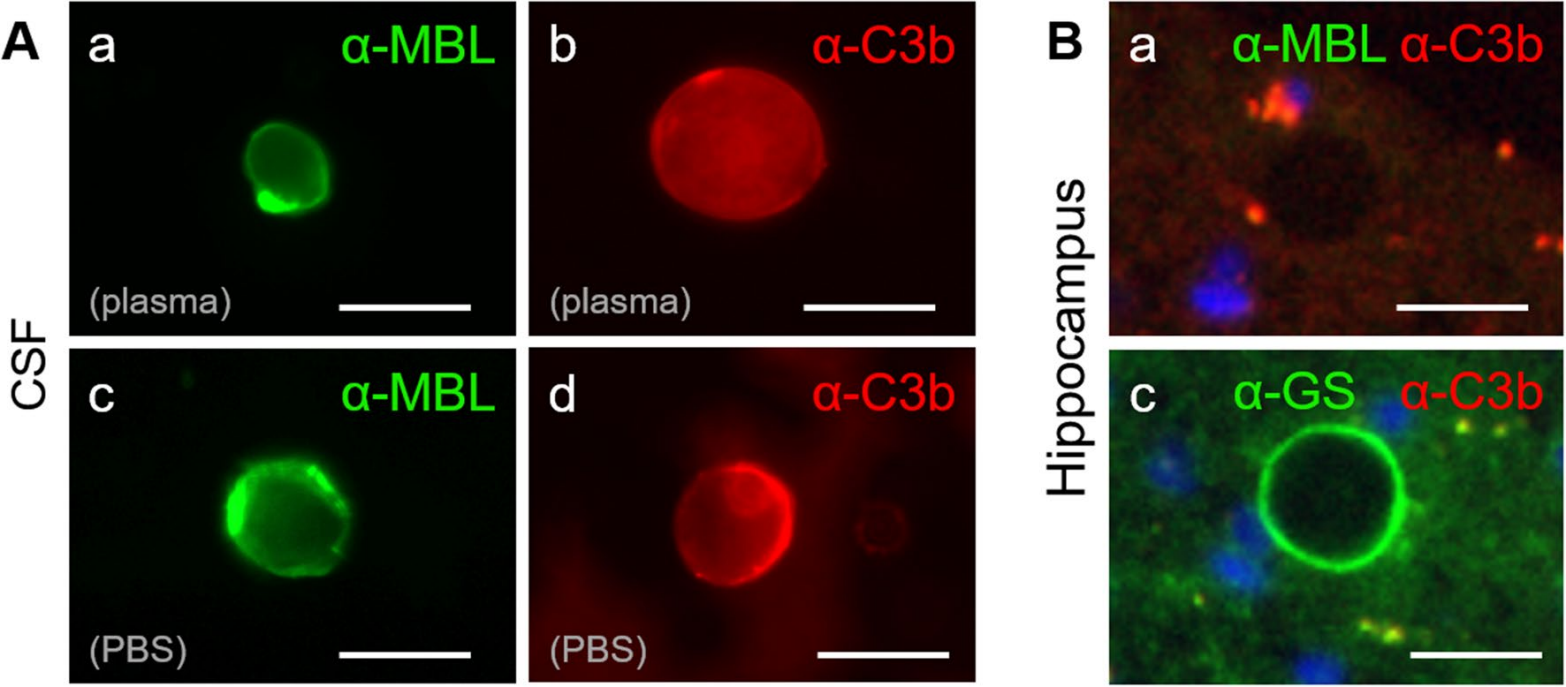
**A** Wasteosomes from CSF have opsonins on their surface. **a** wasteosomes purified from CSF and incubated with human plasma become stained with anti-MBL (green). **b** wasteosomes from CSF and incubated with human plasma become immunostained with anti-C3b (red). **c** wasteosomes purified from CSF and incubated with PBS instead of human plasma also become immunostained with anti-MBL (green). **d** wasteosomes purified from CSF and incubated with PBS instead of human plasma also become immunostained with anti-C3b (red). **B** Hippocampal wasteosomes do not have the opsonins on their surface. **a** When the hippocampal tissue is double-immunostained with anti-MBL (green) and anti-C3b (red), the wasteosomes do not become immunostained and are observed as a black circle. In this case, one wasteosome can be observed in the center of the image. **b** When the hippocampal tissue is immunostained with anti-GS and anti-C3b, wasteosomes become stained with anti-GS (green) but not by anti-C3b (red). Scale bars: 25 µm

Wasteosomes have a polyglucosan structure based on polymerized hexoses, which are mainly glucose [1] although not exclusively. As previously mentioned, MBL binds to several hexoses such as mannose and N-acetylglucosamine, while C3b can bind to glucose [48–50]. Accordingly, we explored the CD35- or complement-mediated phagocytosis and analyzed if the wasteosomes from the CSF could be opsonized by MBL or C3b. After incubating the wasteosomes with human plasma, some aliquots were immunostained with the anti-C3b antibody (directed against C3b) while others were immunostained with the anti-MBL antibody. As shown in Fig. 6A, wasteosomes were stained by both the anti-C3b and anti-MBL antibodies, indicating that wasteosomes are opsonized by MBL and C3b. However, and surprisingly, wasteosomes from the control samples (where the wasteosomes were incubated with PBS instead of human plasma) were also stained with the anti-C3b or anti-MBL antibody, indicating that wasteosomes from the CSF are opsonized by MBL and C3b. It is of interest, as commented in the discussion section, that wasteosomes located in the brain parenchyma are not opsonized by these proteins (Fig. 6B).

### Wasteosomes and macrophage interactions at central nervous system interfaces

**Fig. 7:**
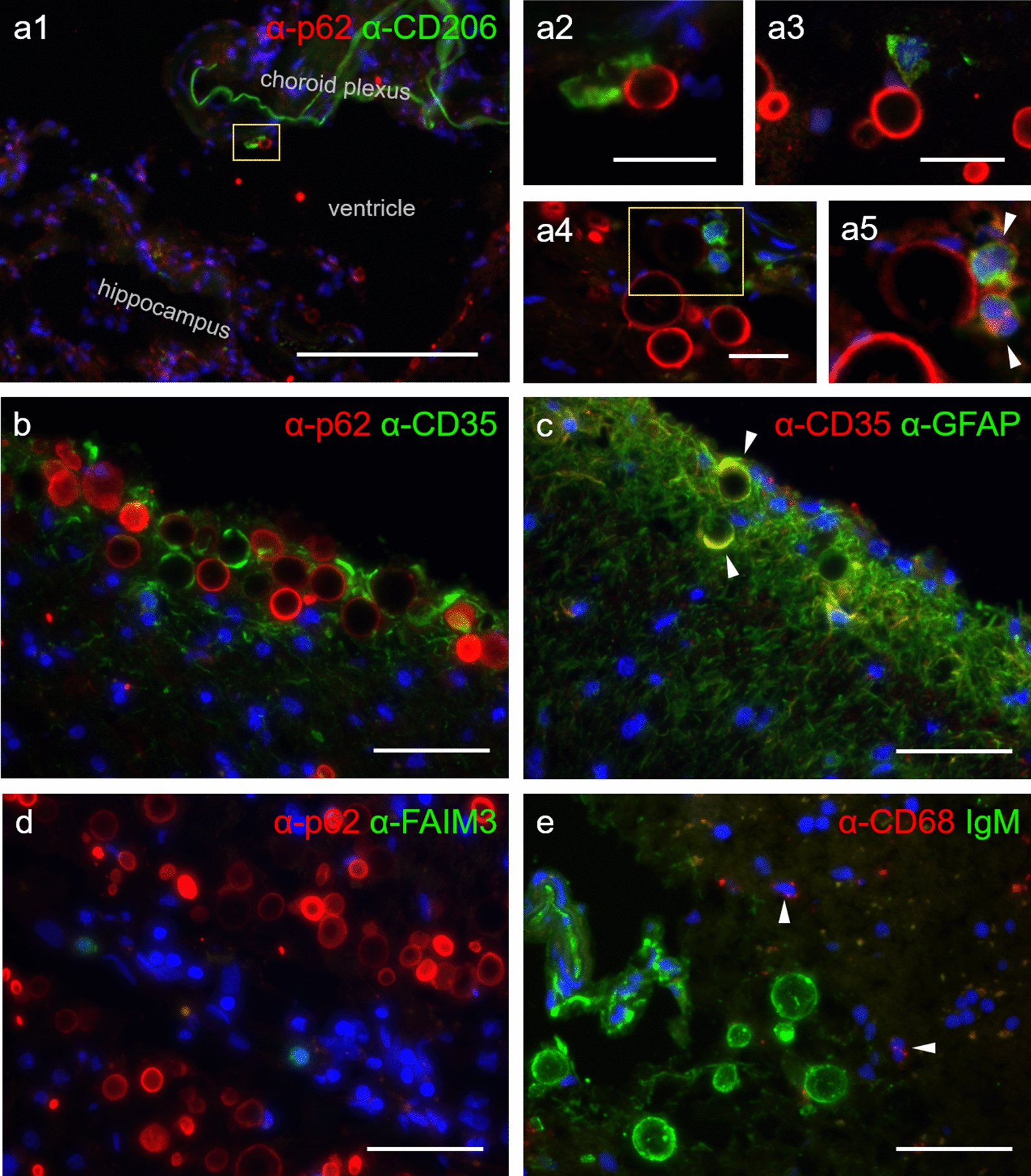
Some macrophages at central nervous system interfaces interact with wasteosomes. **a1**, **a2**, **a3**, **a4** and **a5** wasteosomes from human hippocampal sections immunostained with anti-p62 (red) and interface macrophages immunostained with anti-CD206 (green). **a1** A choroid plexus macrophage in contact with a wasteosome released from the brain tissue to the ventricular CSF. **a2** inset of a1, where the macrophage attached to the wasteosome is magnified. **a3** and **a4** wasteosomes released from hippocampus to the subarachnoid space in contact with meningeal macrophages. **a5** inset of a4, where the red staining is digitally intensified to evidence the presence of a wasteosome, in this case surrounded by two macrophages (white arrowheads). **b** within the brain parenchyma, wasteosomes become immunostained with anti-p62 (red) and are surrounded by CD35 + cells (green). **c** CD35 staining (red) colocalize with GFAP staining (green, white arrowheads), indicating that cells that surround wasteosomes are CD35 + astrocytes. **d** wasteosomes from human hippocampus immunostained with anti-p62. FAIM3 positive cells are not found in the hippocampal sections (green). **e** wasteosomes from human hippocampal tissue immunostained with IgMs (green) are not contacted by CD68 + cells (red, marked with white arrowheads). For their localization, these CD68 + cells are presumably microglial cells, although possible infiltrating macrophages cannot be discarded. In any case, and as expected, microglia or macrophages did not reach the wasteosomes within the brain parenchyma since in this region wasteosomes are intracellular astrocytic structures. Scale bar in a1: 200 µm; scale bars in a2, a3 and a4: 25 µm; other scale bars: 50 μm. Hoechst (blue) was used for nuclear staining

After observing that different mechanisms might be involved in the phagocytosis of wasteosomes by THP-1 macrophages in vitro, we ascertained whether this phagocytosis also happens in vivo when wasteosomes are released from the brain parenchyma into the CSF. As mentioned above, wasteosomes accumulate mainly in the perivascular, periventricular and subpial regions of the brain. When they are expelled from these regions, they may encounter perivascular macrophages, choroid plexus macrophages and meningeal macrophages. Therefore, double immunostaining of human hippocampal sections was performed with the anti-p62 antibody, a protein marker that allows the localization of wasteosomes, together with the anti-CD206, anti-CD35, anti-FAIM3 or anti-CD68 antibody, which are associated with phagocytosis or phagocytic cells. Immunostaining with the anti-CD206 antibody revealed some CD206-positive macrophages that were in contact with wasteosomes. Figure 7a1, exhibiting a section including a part of the hippocampus and the lateral ventricle, shows a CD206-positive choroid plexus macrophage making contact with a wasteosome that has been released from the brain tissue into the CSF in the lateral ventricle. The choroid plexus macrophage attached to the wasteosome can be clearly observed in the magnification of this image shown in Fig. 7a2. Figure 7a3 and 7a4 show several wasteosomes that have been released from the bordering regions of the hippocampus into the subarachnoid space making contact with CD206-positive meningeal macrophages. A magnification of Fig. 7a4 is shown in Fig. 7a5, where the staining of wasteosome is digitally intensified to illustrate the presence of a wasteosome with two encircling meningeal macrophages. Staining with the anti-CD35 antibody also revealed some positive cells located at the border of the brain parenchyma surrounding several wasteosomes (Fig. 7b). As astrocytes in the *glia limitans* of brain cavities can be positive for CD35 [54, 55], we tested the possible colocalization of CD35 with GFAP, which is a specific marker of astrocytes. As shown in Fig. 7c, CD35 staining colocalized with GFAP staining, indicating that the cells containing the wasteosomes are not macrophages in this case, but astrocytes. The staining with the anti-FAIM3 antibody did not show any positive cells in the hippocampal sections (Fig. 7d). Finally, the staining with the anti-CD68 antibody stained some cells located in the brain parenchyma. For their localization, these cells are presumably microglial cells, although it cannot be discarded possible infiltrating macrophages. In any case, these CD68 positive cells do not contact with wasteosomes (Fig. 7e). As expected, microglia or macrophages did not reach the wasteosomes within the brain parenchyma since in this region wasteosomes are intracellular astrocytic structures.

## Discussion

The study of wasteosomes has been intriguing since its inception, mainly due to the enormous variety in the results that have been obtained and the difficulty of fitting them all into a consistent and coherent theory. From this perspective, it is not surprising that the present study, which initiates a new line of research focused on the interaction of wasteosomes with macrophages, also leads to diverse findings and leaves open different possibilities.

In the first part of the present study, involving time-lapse assays, we observed that THP-1 macrophages phagocytose or interact with ConA-, AF555-NHS- and PAS-labeled wasteosomes. We also observed that once phagocytosed, ConA-labeled and AF555-NHS-labeled wasteosomes are digested or fragmented and (at least) the fluorescent protein fraction is exposed on the surface of macrophages as well as transferred from one macrophage to another. We observed a lower reactivity of macrophages towards PAS-stained wasteosomes. Although macrophages also contacted with wasteosomes, we did not observe the fluorescent fraction (in this case, the glycan component) spreading throughout the cytoplasm of the macrophages. This could be attributed to a different processing inside the macrophages of the protein fraction of the wasteosomes with respect to the carbohydrate one. Alternatively, it could be due to the changes generated by the PAS staining in the carbohydrate skeleton of the wasteosomes, which would make the wasteosomes less susceptible to the activities of lysosomal amylases or enzymes. In any case, the reactivity of THP-1 macrophages to wasteosomes was observed in every set of experiments.

**Fig. 8:**
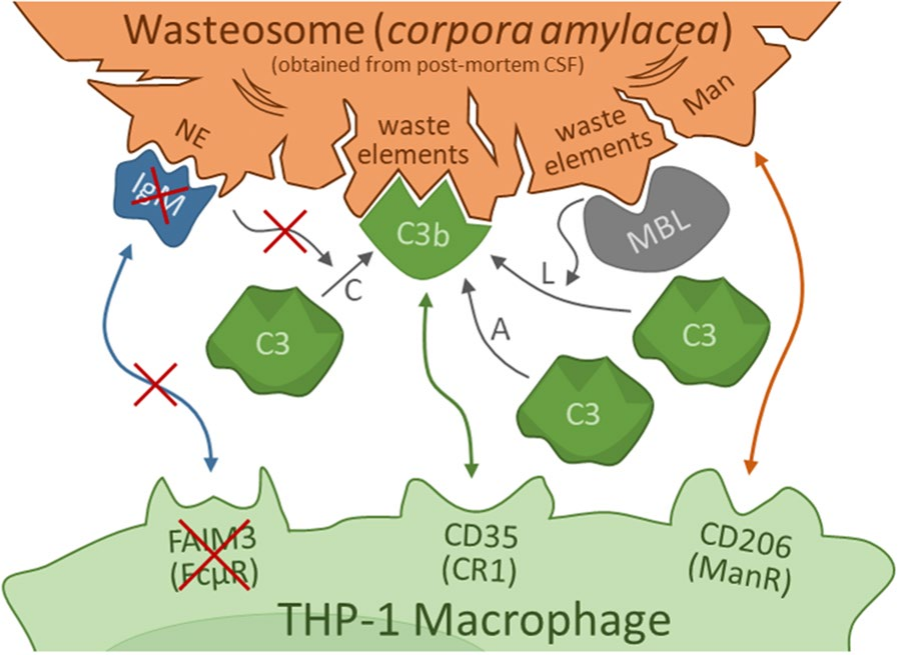
Integrative scheme of the potential mechanisms involved in the phagocytosis of wasteosomes obtained from post-mortem extracted CSF by THP-1 macrophages.The absence of FAIM3 in THP-1 macrophages and the absence of IgM opsonizing the CSF wasteosomes (binding to their NEs) indicate that the phagocytosis by THP-1 macrophages does not occur through the IgM-FAIM3 interaction. However, phagocytosis could occur through CD206 and CD35 receptors, which are both present in THP-1 macrophages. In the first case, the CD206 receptor can interact with mannose or other carbohydrates located in the wasteosomes. In the second one, the CD35 receptor can recognize the C3b that opsonizes wasteosomes once they arrive at the CSF. The opsonization with C3b in the CSF can be produced through the lectin pathway (L), triggered by the binding of MBL with the wasteosomes, and through the alternative pathway (A), triggered by the contact between C3 and wasteosomes, but not through the complement pathway (C), because IgM do not opsonize wasteosomes in the CSF. Note that wasteosomes are obtained from post-mortem extracted CSF and are altered wasteosomes that may show waste elements. At the central nervous system interfaces, in which waste elements are enclosed into the polyglucosan structure, the wasteosomes are not opsonized by MBL or C3b, and they interact with CD206 + macrophages but not with CD35 + or FAIM3 + macrophages. See text for details

In the second part of the study we identified phagocytic receptors on the THP-1 macrophages by analyzing the macrophages that are interacting with wasteosomes, and in the third part we identified the presence of opsonins on the wasteosome surface. Figure 8 displays a broad overview of the results obtained. The absence of FAIM3 in THP-1 macrophages and the absence of IgM opsonizing the CSF wasteosomes indicate that the phagocytosis by THP-1 macrophages does not occur through the IgM-FAIM3 interaction. However, phagocytosis may occur through CD206 and CD35 receptors, which are both present in THP-1 macrophages. In the first case, the target elements of the CD206 receptors in the wasteosomes may be mannose or N-acetylglucosamine residues, since wasteosomes are recognized by ConA, and these residues are targets of both ConA [56–58] and CD206 proteins [45]. In the second case, the CD35 receptor can recognize the C3b that opsonizes wasteosomes. As C3b is not present in tissular wasteosomes, the opsonization with C3b must be produced in the CSF. As CSF contains both C3 and MBL [59–63], in CSF the opsonization with C3b may be produced through the lectin pathway triggered by the MBL that links to wasteosomes, and through the alternative pathway triggered by the contact between C3 and wasteosomes, but not through the complement pathway related with IgMs, because IgMs do not opsonize wasteosomes in the CSF.

After describing some of the possible interactions between wasteosomes and THP-1 macrophages, the fourth part of the study assesses possible interactions between wasteosomes and macrophages located in the central nervous system. In the brain histological sections, and throughout the various experiments, macrophages attached to wasteosomes were only observed at the brain interfaces. The fact that this interaction does not occur in the brain parenchyma is to be expected, since in this location the wasteosomes are inside the astrocytes [40]. Notably, the macrophages that are attached to wasteosomes—for instance, some choroid plexus macrophages and some meningeal macrophages—have been shown to be CD206 + macrophages, but never CD35 + . As mentioned, we observed CD35 + cells encompassing some wasteosomes in the subpial regions, but these cells are CD35 + astrocytes, which might in fact be generating the wasteosomes. These CD35 + astrocytes have been described previously, and although the function of the CD35 + receptors that they present is not known, they have been associated with processes of information exchange between the immune system and astrocytes [54, 55]. Thus, the tissue studies show that the macrophages that interact with wasteosomes are CD206 + but not CD35 + . Coincidentally, in previous studies we observed that the wasteosomes that reach the cervical lymph nodes bind to cells which are compatible with macrophages and do not express CD35 or FAIM3 receptors either [40]. It seems, therefore, that there is some discrepancy between the studies carried out with THP-1 macrophages and the studies of histological sections, since in the former both the CD206 and CD35 receptors may be involved in the phagocytosis of wasteosomes, while in the latter the CD206 receptors may be involved but not the CD35 receptors.

However, there is a difference between the experiments performed with THP-1 macrophages and those performed with brain slices. In the first, the interaction between THP-1 macrophages and wasteosomes was studied by using wasteosomes that were obtained post-mortem from CSF extracted via intraventricular puncture. Prior to the interaction with the THP-1 macrophages, those wasteosomes would have experienced a certain degradation or digestion processes due to the post-mortem delay. During this time, the structure of the wasteosomes may have changed, exposing the waste elements that are included in them such as membranous or organelle remnants, which can trigger the opsonization of the wasteosomes by MBL and C3b in the CSF itself. These altered wasteosomes may therefore be recognized not only by CD206 receptors, but by CD35 receptors as well. In physiological conditions, on the other hand, in which the wasteosomes serve precisely to eliminate waste substances and isolate potentially toxic elements, the residual and potentially inflammatory elements appear to be integrated or encompassed in the polyglucosan structure, thus avoiding the opsonization with MBL or c3b. These wasteosomes can therefore trigger responses mediated by CD206 but not by CD35, explaining why in tissue studies the macrophages attached to wasteosomes are only CD206 + macrophages.

CD206 is normally expressed on the M2 macrophage subtype, but not on M1 [46]. Although the classification of macrophages as M1 or M2 subtypes is not clear or binary [64, 65], it is interesting that M2 macrophages, such as those of the M2a or M2c subsets that contain the CD206 receptors, are considered to be anti-inflammatory, with M2a macrophages being involved in tissue remodeling and M2c macrophages associated with the phagocytosis of apoptotic cells [66–68]. From this point of view and bearing in mind that wasteosomes include waste elements that would need to be removed without triggering unnecessary inflammatory responses, it seems conceivable that under physiological conditions wasteosomes bind to these macrophages through CD206 + , generating non-inflammatory responses. The interaction of CD35 + macrophages and wasteosomes would occur only in cases in which the wasteosomes are altered, thus exposing the waste elements or potentially toxic elements that they contain. In these cases, not only would the wasteosomes be degraded by the macrophages, but also, as observed with THP-1 macrophages, part of the components of the wasteosomes would be exposed on the surface of the macrophages and exchanged between the different macrophages as part of the inflammatory response itself.

## Conclusions

The present study indicates that macrophages have the machinery required to process and degrade wasteosomes, and that macrophages can interact in different ways with wasteosomes. In physiological conditions, the main mechanism involves the M2 macrophages and CD206 receptors, which trigger the phagocytosis of wasteosomes without triggering inflammatory responses, and thus avoiding tissue damage. However, in the case of abnormal or altered wasteosomes, as they expose waste elements or potentially toxic elements, become opsonized by MBL and C3b. Thus, in this case, CD35 receptors constitute another possible mechanism of phagocytosis, leading to inflammatory responses. Accordingly, as wasteosomes export brain substances avoiding the blood brain barrier, we need to establish whether some of the macrophages that phagocytose the wasteosomes participate in the induction of immunological tolerance and of adaptive immunological responses in the central nervous system, and whether they also play significant roles in some brain autoimmune diseases.

## Supplementary Information

**Additional file 1: Video 1A**. Video from a time-lapse recording, showing how a THP-1 macrophage phagocytose a wasteosome opsonized with ConA.

**Additional file 2: Video 1B**. Video showing the location of the remains of a wasteosome opsonized with ConA after being phagocytosed and fragmented by a THP-1 macrophage.

**Additional file 3: Video 2A**. Video from a time-lapse recording, showing two THP-1 macrophages eroding a wasteosome opsonized with ConA.

**Additional file 4: Video 2B**. Video from a time-lapse recording, showing different macrophages interacting with three different wasteosomes opsonized with ConA.

**Additional file 5: Video 3A**. Video from a time-lapse recording, showing a THP-1 macrophage making contact and eroding a wasteosome stained with the AF555-NHS dye.

**Additional file 6: Video 3B**. Video from a time-lapse recording, showing that a THP-1 macrophage erodes a wasteosome stained with the AF555-NHS dye and presents thereafter some spots of fluorescence at their surface.

**Additional file 7: Video 4A**. Video from a time-lapse recording, showing a THP-1 macrophage contacting a wasteosome stained with PAS.

**Additional file 8: Video 4B**. Video from a time-lapse recording, showing THP-1 macrophages contacting and eroding wasteosomes stained with PAS.

## Data Availability

The data sets and materials are available on request to the corresponding author.

## Abbreviations

AF: Alexa fluor
APCs: Antigen-presenting cells
BB: Blocking buffer
BSA: Bovine serum albumin
ConA: Concanavalin A
CR1: Complement receptor type 1
CSF: Cerebrospinal fluid
DPBS: Dulbecco’s PBS
FBS: Fetal bovine serum
FI: Fluorescein
fpm: Frames per minute
GFAP: Glial fibrillary acidic protein
GS: Glycogen synthase
MBL: Mannose-binding lectin
NEs: Neoepitopes
PAS: Periodic acid-Schiff
PBS: Phosphate-buffered saline
PMA: Phorbol 12-myristate 13-acetate
Rhod: Rhodamine
ROIs: Regions of interest
THP-1 macrophages: Macrophages derived from THP-1 cells
